# Vision recovery in human immunodeficiency virus-infected patients with optic neuropathy treated with highly active antiretroviral therapy: A case series

**DOI:** 10.4103/0301-4738.53062

**Published:** 2009

**Authors:** Kalpana Babu, Krishna R Murthy, Nirmala Rajagopalan, B Satish

**Affiliations:** Vittala International Institute of Ophthalmology and Prabha Eye Clinic and Research Center, Bangalore, India; 1 Freedom Foundation, Bangalore, India; 2 Seva Clinic, Bangalore, India

**Keywords:** Highly active antiretroviral therapy, human immunodeficiency virus, optic neuropathy

## Abstract

We describe three patients with bilateral, presumed human immunodeficiency virus (HIV)-induced optic neuropathy. The above diagnosis was made by exclusion of infectious agents and neoplasms by detailed clinical and laboratory investigations. All patients had decreased visual acuity, pale optic discs and constriction of visual fields. Improvement was documented in all three patients for visual acuity and in one patient for visual fields following treatment with highly active antiretroviral therapy (HAART). Optic neuropathy in HIV-positive patients does not necessarily carry a poor prognosis even when a treatable cause is not found. This article emphasizes the effectiveness of HAART in presumed HIV-induced optic neuropathy.

Optic nerve involvement in patients with human immunodeficiency virus (HIV) infection can occur as a result of infections (cryptococcus, syphilis, tuberculosis, toxoplasmosis, herpes group of viruses), neoplasms (lymphoma), toxic effects of drugs (dideoxyinosine, ethambutol) or directly by the HIV virus.[1] The diagnosis of primary optic neuropathy probably due to HIV is thus by exclusion. We present three such cases.

## Materials and Methods

Three patients with primary optic neuropathy probably due to HIV and a minimum follow-up of six months after starting highly active antiretroviral therapy (HAART) were included in this study. All the patients were on a combination of trimethoprim and sulphamethoxazole once daily for *Pneumocystis carinii* prophylaxis. Best-corrected visual acuity (BCVA), extraocular movements, pupillary, anterior segment, intraocular pressures and dilated fundus examinations were done in all patients. Visual fields were assessed in eyes with good vision on Humphrey visual field analyzer (Zeiss, Germany).

The following investigations were done in all patients: Complete blood counts, serum electrolytes, blood sugar, blood and urine cultures for bacteria and fungi, ELISA for toxoplasmosis, *herpes simplex (HSV)1 and 2*, *varicella zoster (VZV)* and *cytomegalovirus (CMV)*, venereal disease research laboratory test (VDRL) and *treponema pallidum* hemagglutination test (TPHA) for syphilis, chest X-ray and Mantoux test for tuberculosis. Cerebrospinal fluid (CSF) was examined for glucose, cells, proteins, bacterial and fungal cultures, ELISA for toxoplasmosis and VDRL for syphilis. CSF smears for acid-fast bacilli (Ziehl Neelsen), Indian ink preparation and culture for *Cryptococcus neoformans*, latex agglutination test for *Cryptococcus* antigen were also done. Polymerase chain reaction (PCR) on the CSF for *HSV, VZV* and *CMV* were done in two cases. Magnetic resonance imaging (MRI) of the orbit and cranium were done to rule out any intracranial space-occupying lesions. In all three patients, the diagnosis of primary optic neuropathy, probably due to HIV, was by exclusion. Visually evoked potential (VEP) was done in one patient. Mitochondrial mutations associated with Leber's hereditary optic neuropathy were not tested for in our patients.

### Case 1

**Figure 1:**
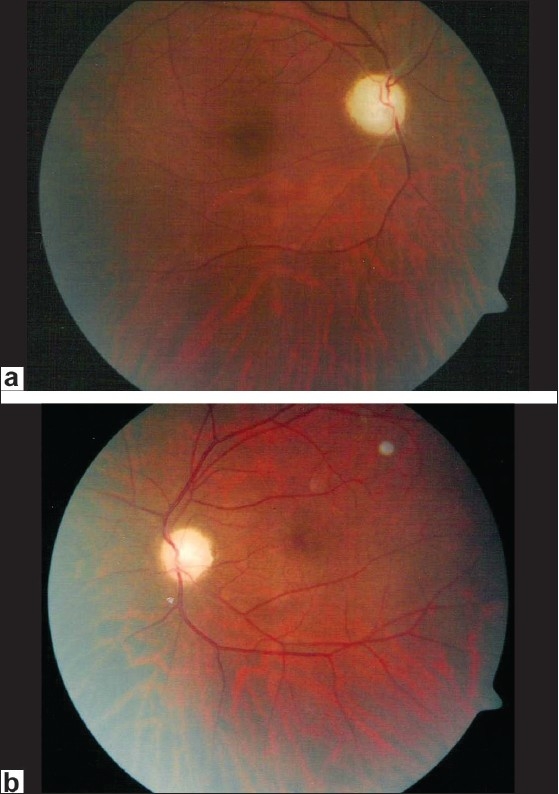
Fundus photograph of case 1 showing optic disc pallor in right (a) and left (b) eye

A 30-year-old retrovirus positive male was referred for a bilateral slowly deteriorating vision over a six-month period. His CD4 and absolute lymphocyte counts at the time of referral were 120 cells × 10^6^/L and 2300 cells/mm^3^, respectively. At the time of presentation, he was on a combination of trimethoprim and sulphamethoxazole. BCVA was counting fingers at 1 meter in both eyes. Old visual fields two months prior to his referral showed constriction in both eyes. The optic discs were pale in both eyes [Figs. 1a and b]. There was no relative afferent pupillary defect. VEP showed decreased amplitude and increased latency in both eyes. At seven months follow-up, following HAART, his BCVA has improved to 20/60 and 20/40 in the right and left eyes respectively. The CD4 count at this follow-up was 210 cells × 10^6^/L.

### Case 2

**Figure 2:**
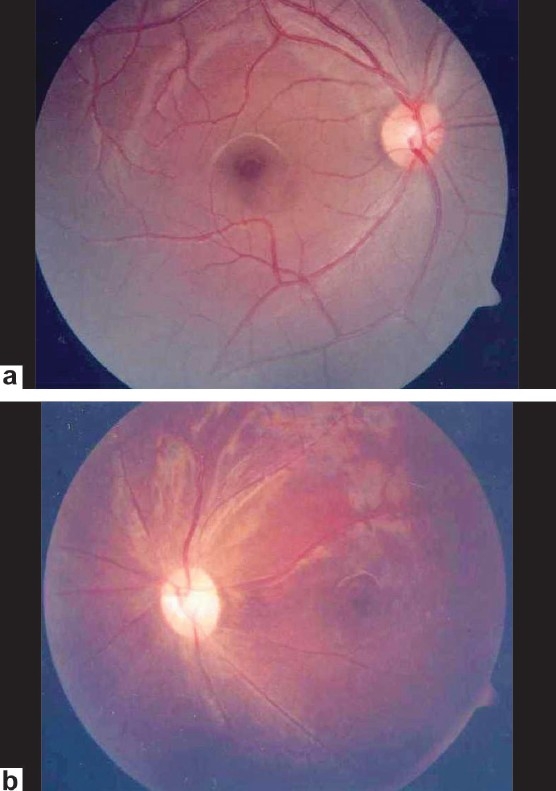
Fundus photograph of case 2 showing mild optic disc pallor in right eye (a) and pale optic disc in left eye (b)

A 13-year-old retrovirus positive girl was referred to us for a routine ophthalmic evaluation. At the time of examination, she was unaware of the poor vision in the left eye. Her BCVA was 20/30 and counting fingers close to the face in the right eye and the left eye, respectively. Relative afferent pupillary defect was noted in the left eye. CD4 counts were 287 cells × 10^6^/L. Mild optic disc pallor in the right eye and pale optic disc in the left eye was noted [Figs. 2a and b]. Visual fields (HVF 30-2) in the right eye showed gross constriction [Figures 3a and b]. After starting HAART, her BCVA at 20 months follow-up is 20/20 in the right eye and counting finger at 3 meters in the left eye. Serial visual fields also showed improvement in the right eye [Figures 4a and b]. The CD4 count at this follow-up was 662 cells × 10^6^/L.

**Figure 3a:**
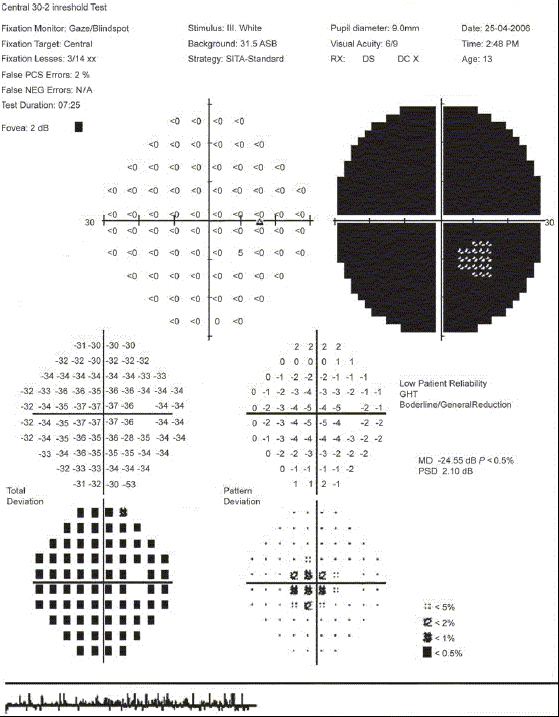
Photograph of the visual fields of the right eye of case 2 showing gross constriction of visual fields, HVF 30-2

**Figure 3b:**
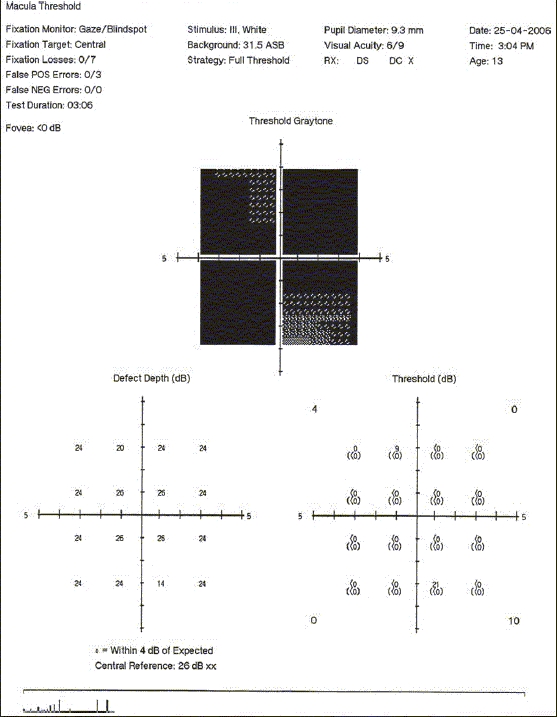
Macular threshold

**Figure 4a:**
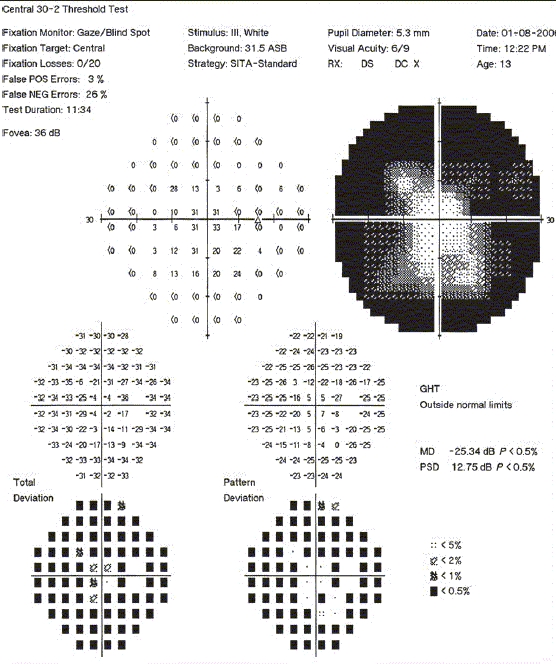
Serial photographs of visual fields of the right eye 4 months after starting HAART

**Figure 4b:**
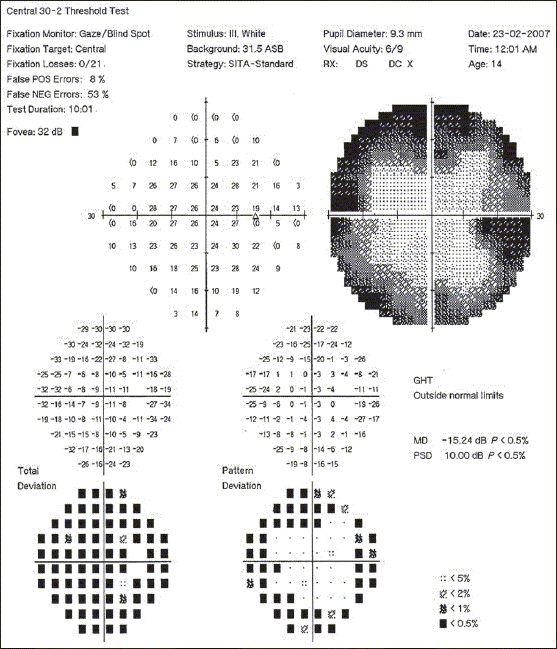
Serial photographs of visual fields of the right eye 10 months after starting HAART

### Case 3

A 31-year-old retrovirus positive male complained of decrease in vision in both eyes. His BCVA in both eyes was hand movements close to face. CD4 count was 189 cells × 10^6^/L. Mild optic disc pallor was noted in both eyes. There was no relative afferent pupillary defect. Visual fields could not be done due to the poor vision. At six months follow-up after starting HAART, his BCVA is 20/200 in both eyes. The CD4 counts were not available at the last follow-up.

## Discussion

Neuro-ophthalmological disturbances have been widely described in both asymptomatic and symptomatic HIV-positive subjects.[2,3] There is sufficient evidence that optic nerves in HIV-infected patients can undergo chronic degeneration and consequent axonal loss. This could be due to infectious, inflammatory and compressive causes.[2,4,5] The mechanism by which HIV induces primary optic neuropathy remains to be clarified. In a study by Sadun *et al.*,[6] patchy axonal degeneration, oligodendrocyte, and myelin degeneration were noted in association with mononuclear cell infiltration, suggesting that optic nerve degeneration may be mediated by HIV-infected macrophages. As the viral particles have never been detected in the optic nerve axons either by electron microscopy or *in situ* hybridization, the current hypothesis favors an immune-mediated mechanism possibly due to the indirect effect of cytokines released by the virus-infected macrophages, particularly TNF-α which plays a major role in HIV-induced neuronal apoptosis.[6,7] In our series, except the HIV infection, there was no other discernible cause of the optic neuropathy. The improvement seen (vision and in serial visual fields) in our patients with HAART is suggestive that much of the optic nerve failure is due to a reversible dysfunction of the optic neurons rather than their death. HAART probably is effective in the initial phases when the axonal degeneration has still not set in. Our series could have been strengthened with electrophysiological studies like pattern electro retinogram (ERG) in documenting evidence of any subclinical retinal dysfunction.[8]

In conclusion, HIV may directly cause an optic neuropathy. Optic neuropathy in HIV-positive patients does not necessarily carry a poor prognosis even when a treatable cause is not found. This article emphasizes the effectiveness of HAART in presumed HIV-induced optic neuropathy.
